# Susceptibility and disease modifier genes in amyotrophic lateral sclerosis: from genetic associations to therapeutic implications

**DOI:** 10.1097/WCO.0000000000001178

**Published:** 2023-06-14

**Authors:** Sean W. Willemse, Michael A. van Es

**Affiliations:** Department of Neurology, UMC Utrecht Brain Center Rudolf Magnus, Utrecht, The Netherlands

**Keywords:** amyotrophic lateral sclerosis, antisense oligonucleotide, ATXN2, C9orf72, clinical trials, FUS, SOD1, UNC13A

## Abstract

Amyotrophic lateral sclerosis (ALS) is a severe disease characterized by the degeneration of motor neurons. Large-scale genetic studies have now identified over 60 genes that are associated with ALS, which in large part have also been functionally characterized. The purpose of this review is to outline how these advances are being translated into novel therapeutic strategies.

**Recent findings:**

The emergence of techniques that allow the specific therapeutic targeting of a (mutant) gene, in particular antisense oligonucleotide therapy (ASOs), have led to the first successful gene therapy for SOD1-ALS and multiple other gene-targeted trials are underway. This includes genetic variants that modify the disease phenotype as well as causal mutations.

**Summary:**

Technological and methodological advances are enabling researchers to unravel the genetics of ALS. Both causal mutations and genetic modifiers are viable therapeutic targets. By performing natural history studies, the phenotype-genotype correlations can be characterized. In conjunction with biomarkers for target engagement and international collaboration, this makes performing gene-targeted trials ALS feasible. The first effective treatment has now been developed for SOD1-ALS and, with multiple studies underway, it seems realistic that more therapies will follow.

## INTRODUCTION

It has long been recognized that there are familial as well as sporadic forms of amyotrophic lateral sclerosis (ALS). Initially, genetic research in ALS mainly focused on identifying causal gene mutations in those pedigrees in which there was an apparent Mendelian pattern of inheritance of the disease [1]. This approach led to the successful identification of a number of genes, such as *SOD1*, *FUS* and *TARDBP*[2–5]. Subsequently, these discoveries facilitated the implementation of diagnostic testing and genetic counselling in clinical practice. This also enabled researchers to introduce these mutations into model organisms such as mice, through which the pathophysiology of the disease could be studied. Furthermore, it permitted the screening of potential novel therapeutic compounds in these systems [6].

Technological advances over the last decades have paved the way for significant advances in our understanding of ALS. Through next-generation sequencing techniques, large-scale genetic association studies became feasible, which has led to a wave of gene discovery in ALS. In the past, the next step after gene discovery would have been to research the relevant mutations in a mouse model. Generating mouse models that recapitulate the phenotype is unfortunately challenging and time consuming. Although mouse models undeniably continue to form an invaluable tool in ALS research, their downside is that they are not a rapid strategy to interrogate gene-specific pathophysiology. The emergence of induced pluripotent stem cell (IPSC) technology and Crispr/Cas9 proved to be a game changer [7^▪^,^8^]. These techniques allow researchers to rapidly generate human-derived motor neurons from patients carrying ALS mutations as well as create isogenic control cell lines. The application of these techniques deliver useful models and can rapidly provide an initial understanding of gene-specific pathophysiology [7^▪^].

Attempts to translate findings from animal models to therapy have largely been focused on pathways, such as excitotoxicity, oxidative stress and inflammation. However, over the course of the last decade, novel techniques, such as antisense oligonucleotides (ASOs), have been developed that allow the specific therapeutic targeting of a (mutant) gene [9,10]. This novel approach has been particularly successful in a different motor neuron disease, spinal muscular atrophy (SMA) [11]. Given that proof of principle has been provided in SMA, there is a sense of hope that these approaches will also deliver effective treatments for other genetic conditions, such as (familial) ALS. Indeed, very recently, the first ASO treatment was shown to be effective for SOD1-ALS and was approved by the FDA [12^▪▪^]. Following this success, there is an increasing number of early-stage clinical trials in ALS targeting specific genes rather than pathways (Table 1). In this review, an overview is provided of the advances in ALS genetics as well as the subsequent translation into gene-targeted trials and effective therapy. 

**Box 1:**
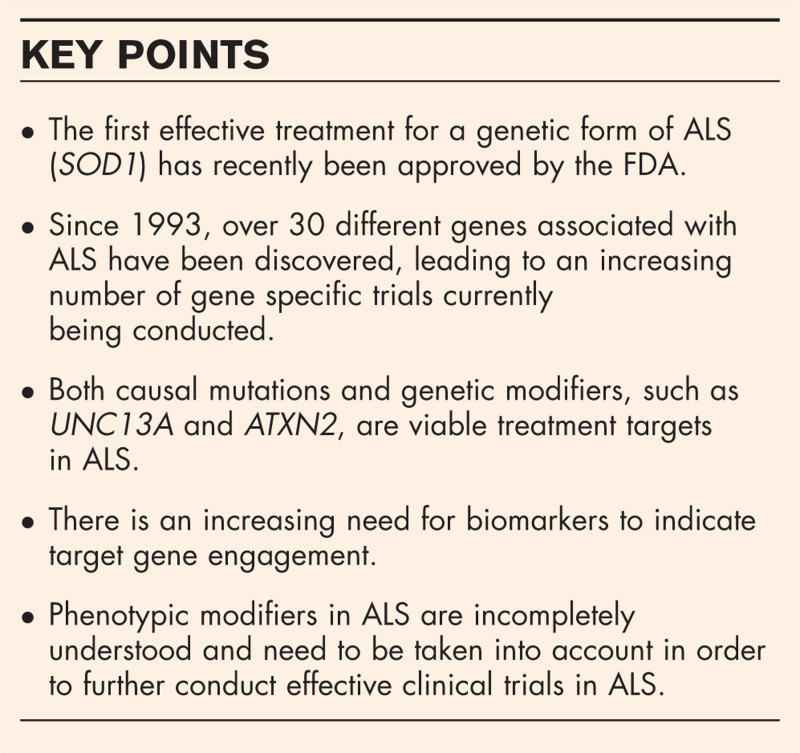
no caption available

**Table 1:** Gene-specific therapeutic trials in amyotrophic lateral sclerosis

				Clinical trials			
Gene	Year of discovery	Year of first targeted trial	Interval from discovery to trial (years)	*n*	Identifier and phase	Year of approval	Interval from discovery to approval (years)
*SOD1*	1993	2010	17	4	NCT01041222; Phase 1	--	--
					NCT01083667; Phase 1/2	--	--
					NCT02623699; Phase 3	2023 FDA (EMA pending)	30
					NCT03070119; OLE		
*FUS*	2009	2021	12	1	NCT04768972; Phase 1–3	--	--
*ATXN2*	2010	2020	10	1	NCT04494256; Phase 1/2	--	--
*C9orf72*	2011	2020	9	5	NCT04288856; Phase 1	--	--
					NCT04931862; Phase 1b/2a	--	--
					NCT04220021: Phase 2	--	--
					NCT05163886; Phase 2	--	--
					NCT04993755: Phase 2	--	--
*UNC13A*	2009	2021	12	1	EudraCT: 2020–000579–19; Phase 3	--	--

EMA, European Medicines Agency; EudraCT, European Union Drug Regulating Authorities Clinical Trials Database; FDA, Food and Drug Administration; OLE, open label extension.

## A BRIEF HISTORY OF AMYOTROPHIC LATERAL SCLEROSIS GENETICS

Given that twin studies have consistently shown that the genetic contribution to the risk of developing sporadic ALS is quite large (estimated at ± 60%), there has always been an interest in performing genetic association studies in ALS patients without a clear family history of the disease. Initially, these types of studies were simply not feasible from a technological point of view. However, novel genotyping techniques were introduced over time, triggering multiple paradigm shifts in ALS genetics.

The first move was from classical linkage analysis in pedigrees to candidate gene studies in sporadic patients. On the basis of existing knowledge of gene/protein function and pathophysiology, genes of interest were selected and the amount of variation within these genes was compared between cases and controls. Despite that at the time this approach was considered to be state-of-the-art, candidate gene approaches were inherently biased and unfortunately did not significantly advance our understanding of ALS.

It proved to be the next generation of techniques that brought the breakthroughs. The introduction of genome-wide association studies (GWAS) facilitated unbiased screens of the entire genome by combining high-throughput genotyping methods and detailed knowledge linkage disequilibrium patterns. In this approach, the association with disease is tested by comparing the frequency of hundreds of thousands of common genetic variants (single nucleotide polymorphisms, SNPs), which tag specific parts of chromosomes that are inherited together (linkage disequilibrium blocks), between cases and controls. Although initially GWAS in ALS had varying success, over time, methodology improved and sample size increased. This allowed the interrogation of both common and rare variants, leading to the finding of robust and replicable novel genetic risk factors for ALS [13,14].

An inherit disadvantage of GWAS is that significant associations do not directly pinpoint actual pathogenic variants, but rather implicate loci that can be very large and contain multiple genes. Therefore, the translation of associations found through GWAS into pathophysiological mechanisms has proven challenging. GWAS hits commonly carry relatively small odds ratios (typically < 1.5) and therefore do not necessarily cause phenotypes when studied in functional experiments.

Moreover, although GWAS in ALS identified multiple novel loci, these hits only explained a small proportion of the genetic risk for the disease. Using GWAS, it is also possible to study the genetic architecture of a disease. This showed that the majority of the genetic risk for ALS is driven by rare variants with intermediate effect size. This means that the genetic risk factors underlying ALS are not as rare and highly penetrant as seen in most Mendelian disease, whilst having higher effect sizes and lower frequencies than most GWAS hits found for common diseases such as hypertension and schizophrenia. Hereby, GWAS defined the search space wherein the missing heritability was hiding and thus paved the way for future genetic research [15].

After GWAS, whole exome sequencing (WES) and even whole genome sequencing (WGS) became feasible and perhaps more importantly affordable. This allowed the final pivot from intelligently selecting markers and thereby inferring where risk factor must lie to ‘simply sequencing everything’. Importantly, WES and WGS are suited to identify rare variants with intermediate effect. Once again, these innovations led to new challenges, such as data storage, methodology for variant calling, statistical power, burden testing and defining thresholds for significance. Nevertheless, WES and WGS fuelled new discoveries, which for instance led to the identification of *TBK1* and the launch of Project MinE [16,17].

Many discoveries were also achieved through combining these different modalities, as was the case for *C9orf72*. Classical linkage analysis had demonstrated that multiple pedigrees shared a very large risk locus on chromosome 9p, in which the pathogenic mutation remained elusive despite large-scale collaborative sequencing efforts. Subsequently, GWAS also identified a locus on chromosome 9p, but which was an order of magnitude smaller. Assuming that these loci overlapped as well as expanding the search to introns, proved to be the essential steps in finding the pathogenic intronic hexanucleotide repeat expansion in *C9orf72*[18–20].

**FIGURE 1:**
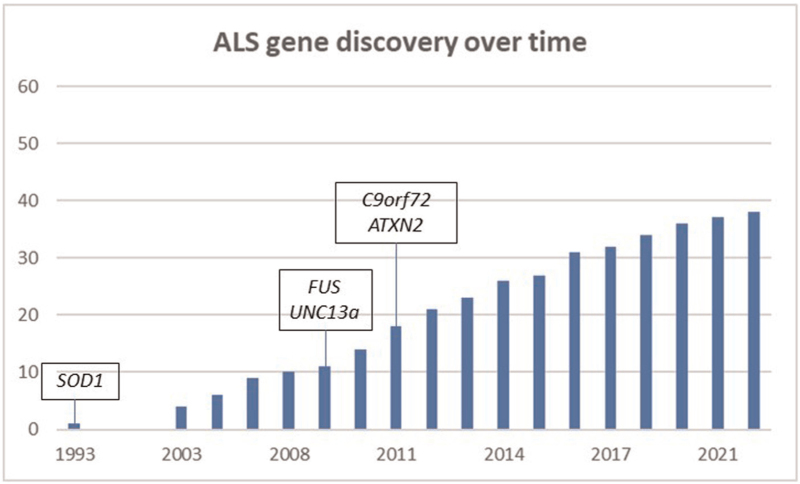
Timeline of gene discovery in amyotrophic lateral sclerosis. Several of the genes mentioned in this review have been highlighted. The x-axis shows the year of discovery. The y-axis shows the total number of identified genes associated with ALS as a running total.

In summary, ALS genetics has undergone multiple revolutions in the last 20 years and has led to a wave of gene discoveries that continue on an exponential pace. To date, over 30 genes have been identified (Fig. 1). Interestingly, the fine-mapping of GWAS hits resulted in associations with familial ALS genes, such as *TBK1* and *SOD1*[15]. Similarly, the use of WES and GWS in (extended) pedigrees and case--control burden analyses have identified the same genes as found by GWAS approaches in which low-frequency variants have been imputed [21]. Lastly, improving methodologies now also permit genome-wide analyses of structural variation such as repeat expansions and the noncoding genome (e.g. cryptic exons or enhancers). Therefore, it seems only a matter of time before remaining genetic risk factors underpinning ALS will be identified.

## FROM GENES TO PATHOPHYSIOLOGY; THE IMPORTANCE OF MODIFIERS

Similar to the technological advances in genetics, the toolbox for studying pathophysiology has also vastly expanded, including approaches such as RNA-seq, clip-seq, IPSCs and Crispr/Cas9. The field also shifted from predominantly relying on transgenic mice to also making use of other model organisms such as *Caenorhabditis elegans*, drosophila and zebrafish. Overall, this has led to tremendous progress in our understanding of ALS. Summarizing these innovations and novel insights falls outside the scope of this review.

However, there are a few insights that stem from the translation of genetics to pathophysiology that we wish to highlight here. ALS genes can be functionally categorized into three broad groups: RNA metabolism, autophagy/proteasome and axonal transport [22]. It seems that pathogenic and risk variants lead to primary defects in these processes, which trigger a downstream chain of events leading to cell stress, the formation of stress granules and subsequent formation of TDP-43 aggregates if these fail to disassemble, prion-like spreading of the aggregates, mitochondrial dysfunction and inflammation. It therefore stands to reason that from therapeutic point of view, the greatest benefit would come from intervening with the main drivers of disease rather than intervening with one of many secondary, downstream effects. The obvious therapeutic targets are therefore causal, pathogenic mutations (large effects with high penetrance). However, an interesting additional category of potential therapeutic targets is formed by phenotypic modifiers with a large effect.

Phenotypic heterogeneity with regards to age of onset, site of onset, degree of cognitive involvement, rate of progression and survival is well recognized within ALS, even within families carrying the same mutation. What drives this variability is incompletely understood. It is thought that both environmental factors (e.g. exposure to common viral pathogens, strenuous physical activity) and additional variation in other genes or loci impact the eventual phenotype.

A clear example of genetic phenotypic modifier with a large effect is the C-allele of the rs12608932 SNP located within the *UNC13A* gene. This SNP was first identified a risk factor through GWAS, but with a very modest effect [odds ratio (OR) < 1.3] [18]. Subsequent studies explored whether GWAS hits also impacted ALS phenotypes, which demonstrated that homozygosity for risk allele of this SNP negatively influences survival by 5–12 months. More in-depth characterization of the *UNC13A* phenotype shows that the age of onset is later (65.5 vs. 63.5 years), the site of onset is more frequently bulbar (43 vs. 31%), frontotemporal dementia is more common (10 vs. 5%), as well lower scores on cognitive screeners such as the ECAS for language and ALS-specific tasks. Imaging studies show that homozygosity for the risk SNP in *UNC13A* is associated with a thinner volume of the right fusiform cortex and the left inferior temporal cortex. Similarly, pathology studies have shown that homozygosity for the risk SNP in *UNC13A* is associated with increased pTDP-43 burden in specific brain areas; middle frontal cortex (8.26 times higher), temporal cortex (4.40 times higher) and motor cortex (3.04 times higher). From this, one can conclude that the C-allele of rs12608932 does not cause ALS, but does profoundly aggravate the phenotype once the disease process has started [23].

Interestingly, two recent groundbreaking studies elucidated what underlies this modifying effect. The intronic region, in which rs12608932 is located, contains a cryptic exon which TDP-43 prevents from being incorporated into mature mRNA under normal conditions. However, under conditions of nuclear depletion (cytosolic mislocalization and aggregation) of TDP-43, this repression is lost, causing the incorporation of the cryptic exon into mRNA and resulting in subsequent nonsense mediated decay and loss of function [24^▪▪^,^25^^▪▪^]. It is this UNC13A loss of function that causes the aggravating effect on the phenotype, as UNC13A performs key functions in synaptic transmission and maintaining neuronal health. Restoring UNC13A expression would therefore seem a reasonable treatment strategy [23].

Another modifier of therapeutic interest are Intermediate polyQ repeats in the *ATXN2* gene [26]. These intermediate repeats are again not causal, but do carry a higher risk for developing ALS. Interestingly, studies have shown that lowering ATXN2 levels leads to a reduction of TDP-43 aggregation. Becker *et al.* demonstrated this by using two independent approaches in mouse models of TDP-43 proteinopathy. First, they created double mutant mice, in which they crossed *ATXN2* knock-out mice withTDP-43 transgenic line. This significantly reduced TDP-43 aggregation and was further accompanied by a dramatic effect on survival and improved motor function. Second, they administered ASOs targeting *ATXN2* to the central nervous system of the TDP-43 mice, which showed similar beneficial effects [27].

## GENE-TARGETED THERAPIES AND TRIALS

Having a thorough understanding of the associated pathophysiology is essential to moving from genetic discoveries to therapeutic application. Fortunately, the interval between these two is becoming ever shorter and with the emergence of ASOs has made gene-targeted treatments and trials a reality. ASOs are short, synthetic, single-stranded oligodeoxynucleotides that have the capability to alter RNA and reduce, restore or modify protein expression through various mechanisms. Although relatively novel, there are multiple FDA-approved ASO therapies for a range of conditions such as Batten disease, Duchenne muscular dystrophy, hereditary transthyretin-mediated amyloidosis, SMA and very recently *SOD1*-ALS.

There are a number of factors which are key when designing a gene-targeted trial. The most crucial of which is having a biomarker that can demonstrate that there is target engagement. This could, for instance, be knockdown or a restoration of the expression of the protein of interest. Safety and data on target engagement are generally used to make go/no go decisions with regards to advancing to the next phase.

In addition, a thorough understanding of the phenotype associated with specific mutations should be a priority. *SOD1*-ALS is associated with considerable phenotypic variability. Certain *SOD1* mutations lead to very aggressive phenotypes (p.A5 V), whereas others (p.D91A) are associated with relatively slow progression [28]. Therefore, the specific mutations that trial participants carry, even within the same gene, will profoundly impact the number of end-points that will occur within the duration of the trial.

The data from the *SOD1* ASO trials suggest that it takes months before clinical benefit becomes apparent. For trials in genetic subforms associated with rapid progression, such as FUS-ALS, this means that it is crucial to enrol patients as early in the disease course as possible in order for the drug to have the maximum chance of being successful. Therefore, inclusion criteria and recruitment strategies should take these aspects into account [29^▪^].

Skewed distributions of mutations associated with either fast or slow progression between the verum and placebo arm might yield incorrect results if not accounted for. There are geographic differences with regards to the prevalence of specific genetic subforms of ALS. *C9orf72* repeat expansions are the most common cause of ALS in populations of European ancestry, but are very rare in populations of Asian descent [30].

## CONCLUSION

The genetics of ALS are being unravelled at an ever-increasing pace and, in combination with our ability to functionally characterize these genetic variants, is greatly advancing our understanding of ALS. The emergence of ASOs has enabled to target genetic variants for therapy. Not only causal gene mutations, but also phenotypic modifiers with a large effect should be seen as viable therapeutic targets. In order to perform a successful gene-targeted trial, it is crucial to have a biomarker for target engagement as well as a firm understanding of genotype-phenotype correlations.

Natural history studies can provide key data on genotype-phenotype correlations (range in age of onset, site of onset, survival, variability biomarker levels in the absence of a therapeutic compound, frequency and geographical distribution of gene mutations and so on). All of these factors profoundly impact critical aspects of trial design, such as which patients (or mutations) to enrol, inclusion criteria, recruitment strategy, trial duration, selection of primary and secondary outcome measures, and so on. Given that many mutations are relatively rare, both natural history studies and clinical trials will require extensive, international collaboration if they are to be successful. Fully acknowledging that are many hurdles to overcome, the track record gives reason for hope that more effective gene-targeted will follow in the near future.

## Acknowledgements

*None.*

### Financial support and sponsorship

*M.A.vE. receives funding support from the Netherlands Organization for Health Research and Development (Vidi scheme), The Thierry Latran Foundation, Motor Neurone Disease Association, FIGHT-MND and the ALS Foundation Netherlands. He is also a member of the European Reference Network for Rare Neuromuscular Diseases (ERN-NMD). S.W.W. is also funded through the Netherlands Organization for Health Research and Development (Vidi scheme).*

### Conflicts of interest

*M.Av.Es. consulted for Biogen, and has received travel grants from Shire (formerly Baxalta), performs work as a medical monitor for an ongoing trial from Ferrer (NCT05178810).*

## REFERENCES AND RECOMMENDED READING

Papers of particular interest, published within the annual period of review, have been highlighted as:

▪ of special interest
▪▪ of outstanding interest
